# Efficacy and safety of venetoclax combined with hypomethylating agents for relapse of acute myeloid leukemia and myelodysplastic syndrome post allogeneic hematopoietic stem cell transplantation: a systematic review and meta-analysis

**DOI:** 10.1186/s12885-023-11259-6

**Published:** 2023-08-17

**Authors:** Yufeng Du, Chunhong Li, Zhijia Zhao, Yikun Liu, Chengtao Zhang, Jinsong Yan

**Affiliations:** 1 Department of Hematology, Dalian Key Laboratory of hematology, Liaoning Medical Center for Hematopoietic Stem Cell Transplantation, the Second Hospital of Dalian Medical University, Dalian, 116027 China; 2 Blood Stem Cell Transplantation Institute, Liaoning Key Laboratory of Hematopoietic Stem Cell Transplantation and Translational Medicine, Dalian Medical University, Dalian, 116044 China; 3 School of Public Health, Dalian Medical University, Dalian, 116044 China; 4 Department of Pediatric, Pediatric Oncology and Hematology Center, Diamond Bay institute of Hematology, Second Hospital of Dalian Medical University, Dalian, 116027 China

**Keywords:** Acute myeloid leukemia, Myelodysplastic syndrome, Venetoclax, Hypomethylating agents, Allogeneic hematopoietic cell transplantation, Relapse

## Abstract

**Background:**

Currently, there is no standard treatment for managing relapse in patients with acute myeloid leukemia and myelodysplastic syndrome (AML/MDS) after allogeneic hematopoietic cell transplantation. Venetoclax-based therapies have been increasingly used for treating post-transplantation relapse of AML. The aim of this systematic review and meta-analysis was to evaluate the efficacy and adverse events of Venetoclax combined with hypomethylating agents (HMAs) for AML/MDS relapse post-transplantation.

**Methods:**

We searched PubMed, Web of Science, Excerpta Medica Database, Cochrane Library, and Clinical. gov for eligible studies from the inception to February 2022. The Methodological Index for Non-Randomized Studies was used to evaluate the quality of the included literatures. The inverse variance method calculated the pooled proportion and 95% confidence interval (CI).

**Results:**

This meta-analysis included 10 studies involving a total of 243 patients. The pooled complete response and complete response with incomplete blood count recovery rate of Venetoclax combined with HMAs for post-transplantation relapse in AML/MDS was 32% (95% CI, 26-39%, I^2^ = 0%), with an overall response rate of 48% (95% CI, 39-56%, I^2^ = 37%). The 6-month survival rate was 42% (95% CI, 29-55%, I^2^ = 62%) and the 1-year survival rate was 23% (95% CI, 11-38%, I^2^ = 78%).

**Conclusion:**

This study demonstrated a moderate benefit of Venetoclax in combination with HMAs for patients with relapsed AML/MDS post-transplantation (including those who have received prior HMAs therapy), and may become one of treatment options in the future. Large-scale prospective studies are needed to confirm the potential benefit from venetoclax combined with HMAs.

**Supplementary Information:**

The online version contains supplementary material available at 10.1186/s12885-023-11259-6.

## Introduction

Acute myeloid leukemia (AML) as a group of complex hematopoietic malignancies is often characterized by adverse molecular biological features and an unfavorable clinical prognosis [1, 2]. Myelodysplastic syndromes (MDS) are a heterogeneous group of clonal hematopoietic stem cell neoplasms that are characterized by ineffective hematopoiesis and a high risk of transformation into AML. The overall prognosis of MDS is poor, especially for high-risk MDS, with a survival time shorter than 1 year [3, 4]. Allogeneic hematopoietic stem cell transplantation (Allo-HSCT) is one of the most promising options for treating AML/MDS. However, relapse remains one of the most critical factors leading to the failure of allo-HSCT [5, 6]. The 2-year survival rate is fewer than 20% for about 30 − 40% of patients with myeloid malignancies relapse after transplantation, especially in these patients with a poor prognosis and an early relapse [7–9].

There is no standard regimen for the treatment of post-transplantation relapse due to the disease heterogeneity, fitness of a patient, ways of transplantation, time to post-transplantation relapse, etc. At present, post-transplantation relapse treatment options primarily include supportive care, withdrawal of immunosuppression, donor lymphocyte infusion (DLI), intensive chemotherapy (IC) ± DLI, hypomethylating agents (HMAs) therapy, and secondary allo-HSCT [10–13]. Among the treatment regimens mentioned above, patients either have unfitness for receiving intensive chemotherapy or secondaryallo-HSCT, or patients with hematological relapse have poor efficacy for DLI and HMAs treatment. Hence, novel therapeutic targets is urgent and necessary to be explored for the relapse of AML/MDS post-transplantation. Venetoclax, a selective BCL2 inhibitor which was approved in 2018 by Food and Drug Administration (FDA), is used in elderly or ineligible patients with newly diagnosed AML,. Venetoclax combined with HMAs (Ven-HMAs) has been gradually adopted to show favorable results in relapsed/refractory AML (R/R AML) and MDS [14–17]. Notably, a minority of the enrolled patients were relapsed AML/MDS post-transplantation who presented effectiveness of Ven-HMAs initially demonstrated by these clinical studies Therefore, Ven-HMAs therapy for treating relapse in AML/MDS post-transplantation has been gradually emerged.

Due to the effect of Ven-HMAs in relapse of patients with AML/MDS post-transplantation has been reported but only on a small scale, leading to an undetermined efficacy and safety, therefore, we aimed to conduct a systematic review and meta-analysis to assess the efficacy and adverse events (AEs) of Ven-HMAs in relapse of patients with AML/MDS post-transplantation.

## Materials and methods

### Data sources and literature search strategy

This meta-analysis has been conducted in the International Prospective System of Register of systematic reviews (PROSPERO) (Registration No. CRD42023398349). The present work was conducted by six researchers. PubMed, Web of Science, Embase, Cochrane Library, and ClinicalTrials.gov were systematically searched from inception to February 2023, and the language was limited to English. The subject terms of this study were “acute, myeloid, leukemia”, “myelodysplastic syndrome”, “venetoclax”, “hematopoietic stem cell transplantation”, “azacitidine”, “decitabine”, and “recurrence”. The complete search strategy is shown in Table S1.

### Literature inclusion and exclusion criteria

Inclusion criteria: (1) Study population: Adult AML/MDS patients who relapsed after transplantation; (2) Intervention: Ven-HMAs regimens; (3) Comparison: IC ± DLI group, DLI group, supportive care group or single arm study; (4) Outcome: The primary outcome indicators were complete response (CR)/complete response with incomplete blood count recovery (CRi) rate, overall response rate (ORR), and survival rate; the secondary indicators were grade 3–4 hematological AEs and incidence of infection; (5) Study design: randomized controlled trial (RCT), retrospective study, prospective study; (6) The number of participants in each study was more than 10 patients.

Exclusion criteria: (1) Case reports, reviews, meta-analysis, commentaries, and non-human studies; (2) Treatment regimens including agents other than Venetoclax in combination with HMAs. (3) Study results without primary outcome indicators; (4) Duplicate publication of literature.

Three investigators (YFD, CHL, and ZJZ), independently read the title, abstract, and full text to decide whether to include a study or not. Any discrepancy in included literatures was resolved by consensus or by consulting another senior investigator.

### Definition of treatment response

We selected included CR/CRi rate, ORR, and survival rate as primary outcome indicators. ORR included the rate of CR/CRi, morphologic leukemia-free status (MLFS), partial remission (PR), and stable disease (SD). Survival rate was defined as the percentage of treated patients who survived after a period of follow-up. The response criteria referred to the 2003 International Working Group, 2017 European Leukemia Network (ELN-2017), and the International Response Unified Criteria revised by the MDS International Working Group in 2006 [18–20].

Grade 3–4 hemocytopenia and incidence of infection were secondary outcome indicators in this research. The different degrees of AEs were rated according to the National Cancer Institute Common Terminology Standard for Adverse Events VERSION 4.03 [21].

### Article quality evaluation

This study assessed the risk of bias using the Methodological Index of Non-Randomized Studies (MINORS) guidelines [22]. Among a total of 12 items in MINORS, the first 8 items are related to non-comparative studies, and the rest 12 items are related to comparative studies. These items were scored as 0 (not reported), 1 (reported but inadequate), or 2 (reported but adequate). Three investigators (YFD, CHL, and ZJZ) independently evaluated the risk bias of the included studies, and any discrepancy was resolved by consensus.

### Data extraction and statistical analysis

Data were independently collected by three investigators (YFD, CHL, and ZJZ) according to a pre-designed Excel sheet. The discrepancy was resolved by consensus or consultation with senior collaborators. R version 4.3.1 software was utilized in the meta-analysis. Analysis of dichotomous variables was performed for dichotomous information CR/CRi, ORR, and AEs using generalized inverse variance without a control group. A 95% confidence interval (CI) was reported for each measure. Q-test and I^2^ test (I^2^ ≤ 25%: no heterogeneity; 26–50%: low heterogeneity; 51–75%: moderate heterogeneity, and > 75%: high heterogeneity) were used for heterogeneity analysis. p < 0.1 or I^2^ > 50% indicated a statistical heterogeneity among references, and a random-effects model was used. When p > 0.1 or I² < 50%, a fixed-effects model was employed for analysis [23]. Subgroup analysis or sensitivity analysis was carried out for results with high heterogeneity to determine the cause of heterogeneity. The sensitivity analysis was performed to evaluate the effect of each study on the statistical results by excluding individual studies one by one.

## Results

### Literature search results

**Fig. 1:**
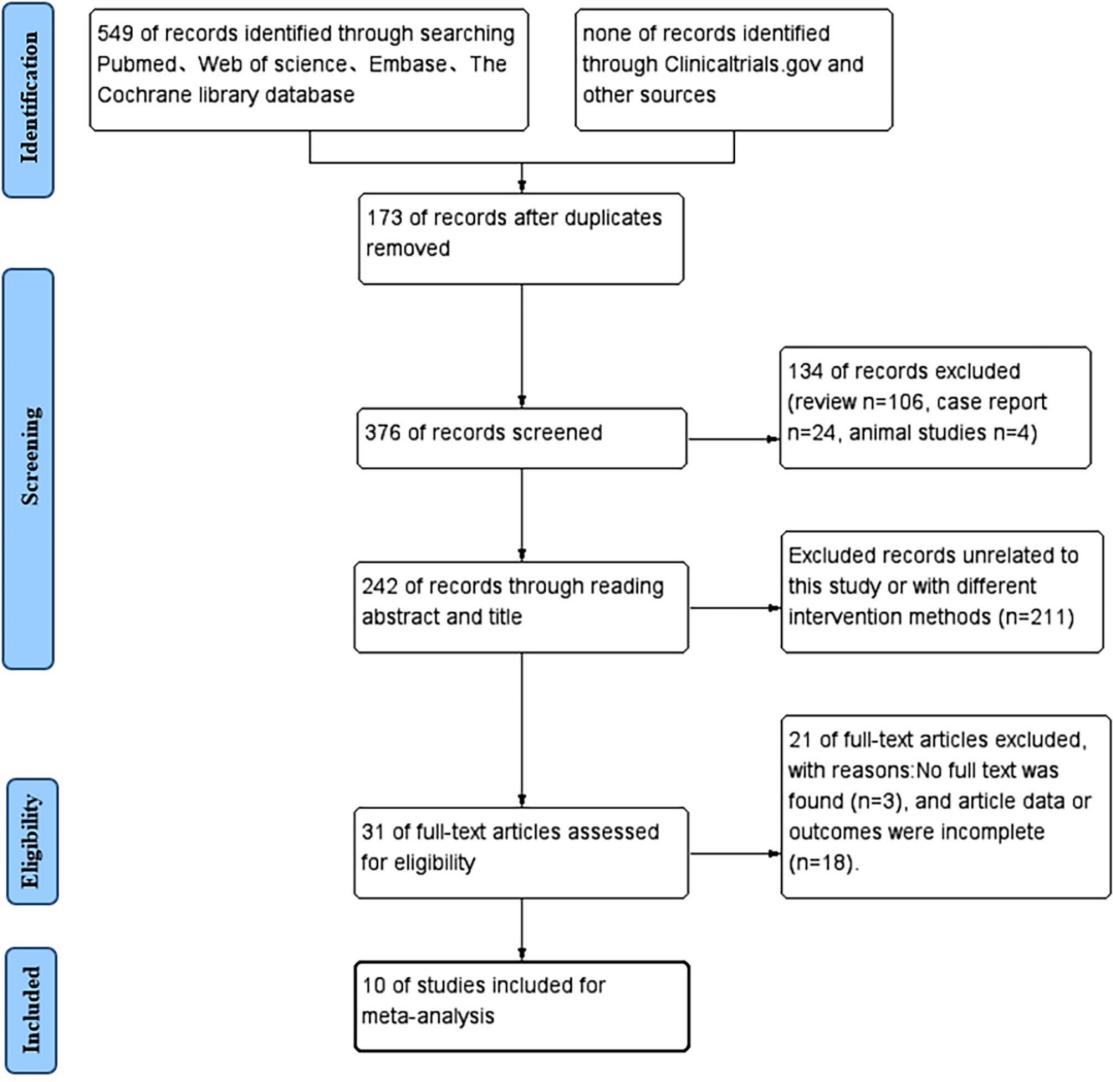
PRISMA flow chart of study selection process

A total of 549 articles were retrieved. Specifically, 173 duplicates and 317 unrelated publications were excluded. Upon reviewing the remained 31 studies in full text, 21 were further eliminated for data unavailability or a lack of primary outcome. The final 10 studies met for the inclusion criteria [24–33]. The literature screening flow chart was prepared using Revman software (shown in Fig. 1).

### Characteristics of the included studies

In total, 10 studies were included in this meta-analysis, all of which were retrospective studies. All the studies included were published from 2019 to 2022. Five studies were based in America, and the other 4 was based in Germany, Italy, Turkey, and China, respectively. Eight studies reported CR/CRi and ORR, respectively. Six studies reported 6-month and 1-year survival rates. AEs were reported in four studies. The included studies are presented in Table 1.

**Table 1:** Clinical characteristics of patients and characteristics of studies included in the meta-analysis

Study	Country	Study type	Diagnose	Patients (numbers)	Male (%)	Median age (years)	Median time to relapse post-Transplantation (months)	Risk stratification by ELN-2017	prior HMAs exposure (%)	Treatment protocol	Efficacy (%)				Adverse effects (3/4) (%)			
											CR/CRi (%)	ORR(%)	median OS (months)	Survival rate (%)	Thrombocyt- openia	Neutropenia	Neutropenic fever	Infection
Mittal. et al. 2019 [24]	American	retrospective	AML	11	NM	66 (25–75)	7 (3–36)	NM	82	Ven + AZA (73%); Ven + DEC (27%) Ven + HMAs + DLI (9%) Ven ≥ 14days/cycle; cycles: 3 (1–20)	36.4	82	11.0	6 months: 81.8 12 months: 36.4	NM	NM	NM	NM
Byrne. et al. 2020 [25]	American	retrospective	AML	16	NM	64.3 (34.5–73.7)	5.7 (0.9–44.9)	Favorable: 6.25% Intermediate: 50% Adverse: 43.75%	NM	Ven + AZA (75%); Ven + DEC (25%) Ven: 14days, 21days or 28days/cycle cycles: 3 (1–11)	31.3	56.3	4.3 (2.1–6.5)	6 months: 18.8 12 months: 6.3	NM	NM	NM	56.3
Diab.et al 2020 [26]	American	retrospective	AML	17	53	62 (31–71)	6.03 (1.5–28.4)	Favorable: 12% Intermediate: 30% Adverse: 58%	35	Ven + AZA (53%); Ven + DEC (47%) cycles: 2 (1–10)	35.3	NM	12.0	NM	NM	NM	47.1	NM
Bewersdorf. et al. 2021 [27]	American	retrospective	AML/MDS	33	51	62 (30–73)	≤ 12 months (70%) ≥ 12 months (30%)	Favorable: 0 Intermediate: 24% Adverse: 76%	61	Ven + AZA (79%); Ven + DEC (21%) Ven + HMAs + DLI (21.6%) cycles: NM	NM	36.4	4.7 (3.8-NR)	6 months: 48.5 12 months: 42.4	NM	NM	NM	NM
Joshi.et al 2021 [28]	American	retrospective	AML	26	48	58 (20–72)	9.0 (2.0–37.0)	Favorable: 3% Intermediate: 35% Adverse: 62%	41	Ven + AZA (31%); Ven + DEC (69%) cycles: 1.5 (1–10)	26.9	38.5	2.6 (1.1–4.1)	6 months: 26.9 12 months: 11.5	65.5	69.0	NM	55.2
Schuler. et al. 2021 [29]	Germany	retrospective	AML/MDS	32	50	54 (30.8–71.5)	1.8 (0.8–42.9)	Favorable: 13% Intermediate: 20% Adverse: 67%	66	Ven + AZA (37.5%); Ven + DEC (59.4%) Ven + AZA + DEC (3.1%) Ven + HMAs + DLI (34.4%) Ven: 21days or 28days/cycle; cycles: 2 (1–19)	31.3	43.8	3.7 (2.8–4.6)	6 months: 37.5 12 months: 21.9	81.3	96.9	NM	71.9
Gao.et al 2021 [30]	China	retrospective	AML/MDS	41	54.5	44 (14–60)	< 12 months(63.6%) > 12 months (36.4%)	others 47.7% adverse 52.3%	NM	Ven + AZA (93.2%); Ven + DEC (6.8%) Ven + HMAs + DLI (2.3%) Ven: 21days or 28days/cycle; cycles: 2 (1–19)	34.1	38.6	NM	6 months: 36.4 12 months: 6.8	68.2	79.5	NM	47.7
Ozturk. et al. 2022 [31]	Turkey	retrospective	AML	30	46.7	43.1 (20–69)	8.5 (1.7–47.8)	Favorable: 6.7% Intermediate: 66.7% Adverse: 26.7%	30	Ven + AZA (93%); Ven + DEC (7%) cycles: 3 (1–6)	43.5	56.5	5.3 (2.6 ~ 8.0)	NM	78.3	82.6	NM	NM
Serpenti. et al. 2022 [32]	Italy	retrospective	AML	11	54.5	65 (31–72)	6.5 (0.9–48.1)	Favorable: 27.3% Intermediate: 45.4% Adverse: 27.3%	73	Ven + AZA (91%); Ven + DEC (9%) Ven + HMAs + DLI (45.5%) cycles: 3 (1–6)	27.3	36.4	NM	NM	NM	NM	NM	27.3
Zhao. et al. 2022 [33]	China	retrospective	AML	26	57.7	35.2 ± 11.4	7.6 (3.2–18.4)	Favorable: 0 Intermediate: 69.2% Adverse: 30.8%	50	Ven + AZA + DLI (100%) cycles: 6–8	26.9	61.5	9.5 (2.7–20.3)	6 months: 53.8 12 months: 50.0	100	100	57.7	NM

Abbreviations: AML, Acute myeloid leukemia; MDS, Myelodysplastic syndromes; ELN, European LeukemiaNet; AZA, azacytidine; DEC, decitabine; HMAs, hypomethylating agents; DLI, Donor lymphocyte infusion; NM, not mentioned;

### Clinical characteristics of included patients

A total of 243 patients were included in the analysis (Table 1). The patients included 230 patients (94.7%) with AML in this meta-analysis. With a sample size of each study ranging from 11 to 41 patients (median size: 26) and an age range of 20-73.7 years old, the proportion of countable males in the trials was 51.7%. This study used ELN-2017 criteria for risk stratification on the included population and suggested that 92.1% of the patients had an intermediate-high risk (intermediate risk: 41.3%, high risk: 50.8%). The morphological/ hematological relapse was observed in 97.5% of patients. In this study, 25.0% of the patients were treated with a combination of DLI. An estimated 52.7% of patients were previously exposed to HMAs.

### Quality assessment of the included studies

The 10 included studies were assessed using the MINORS tool sheet. As all the studies were single-group rate studies, we used the first 8 items for assessment. The quality of evaluation score for each included literature is 12, as shown in Table S2.

### Efficacy of the study

**Fig. 2:**
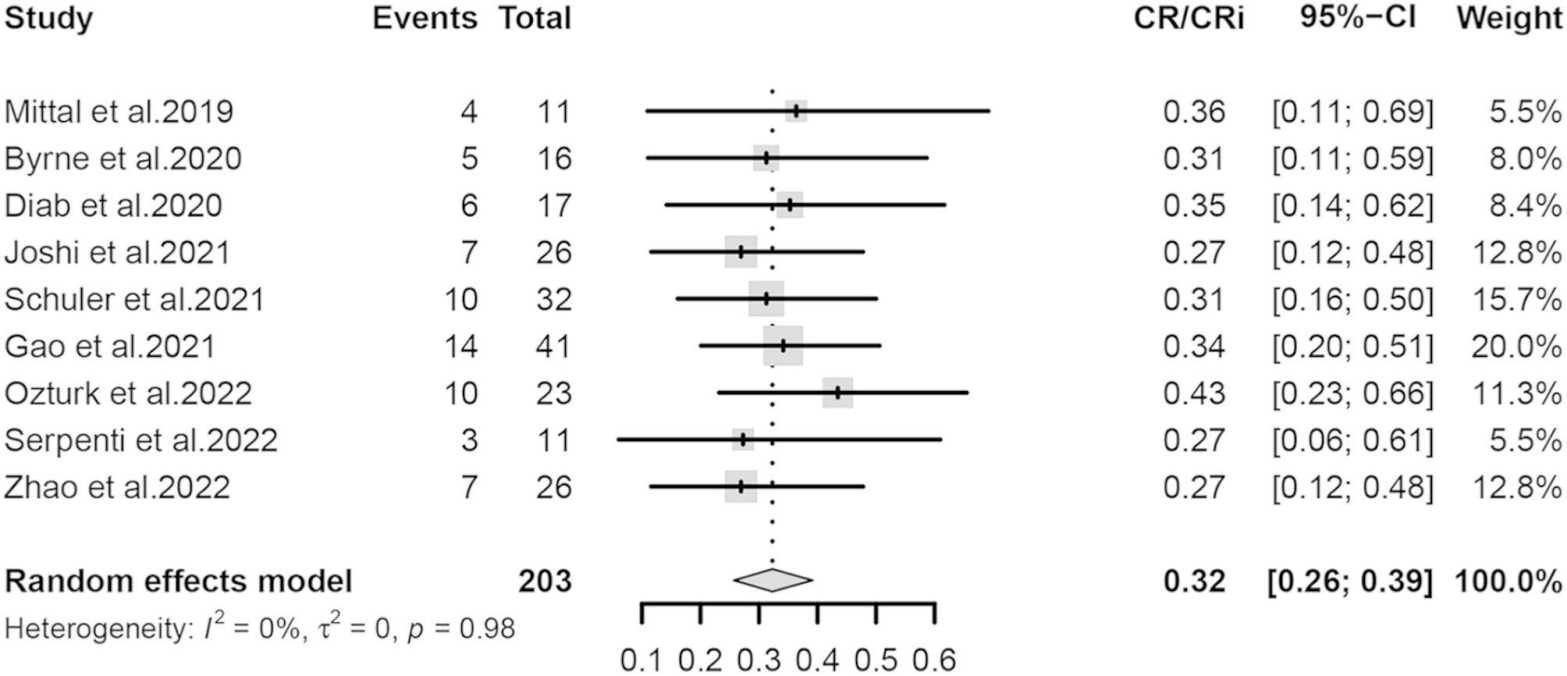
Forest plot of pooled CR/CRi rates after treatment

**Fig. 3:**
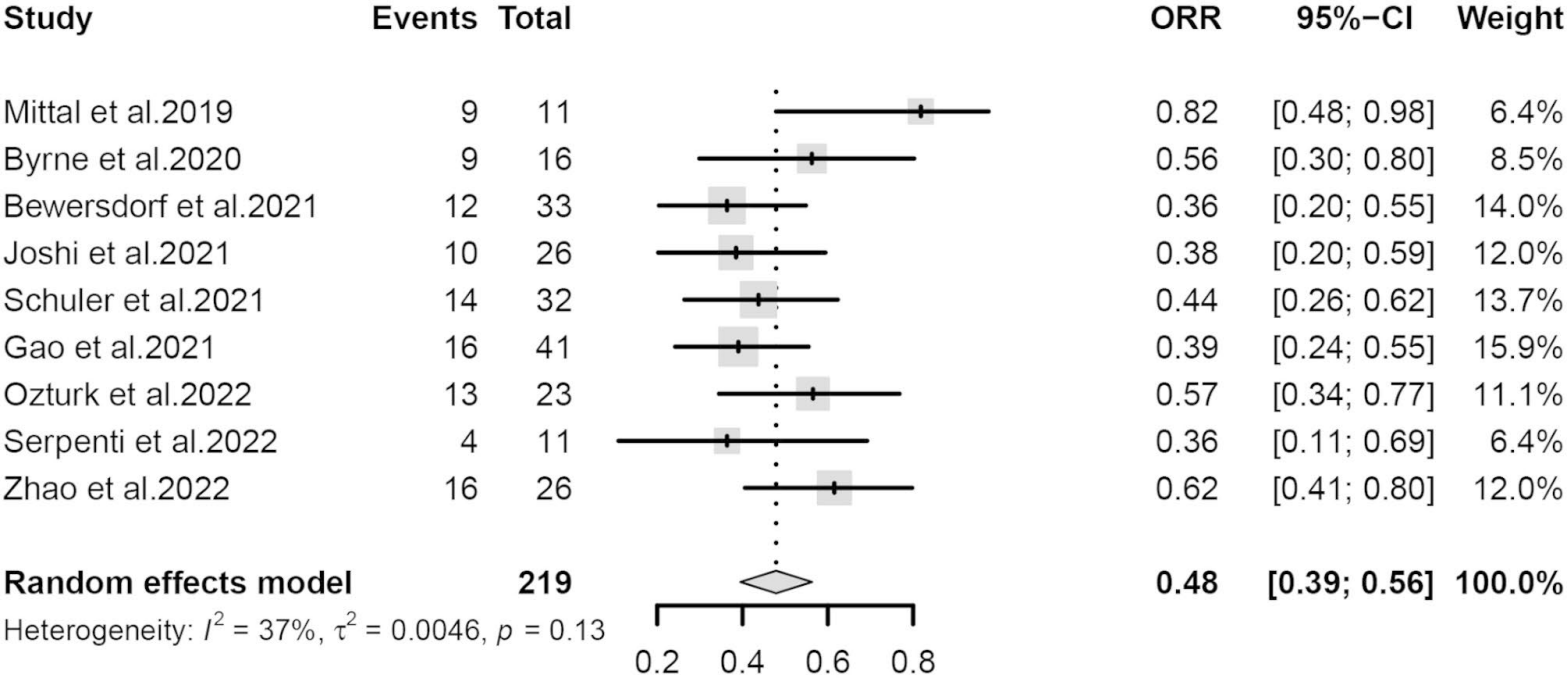
Forest plot of pooled ORR rates after treatment

**Fig. 4:**
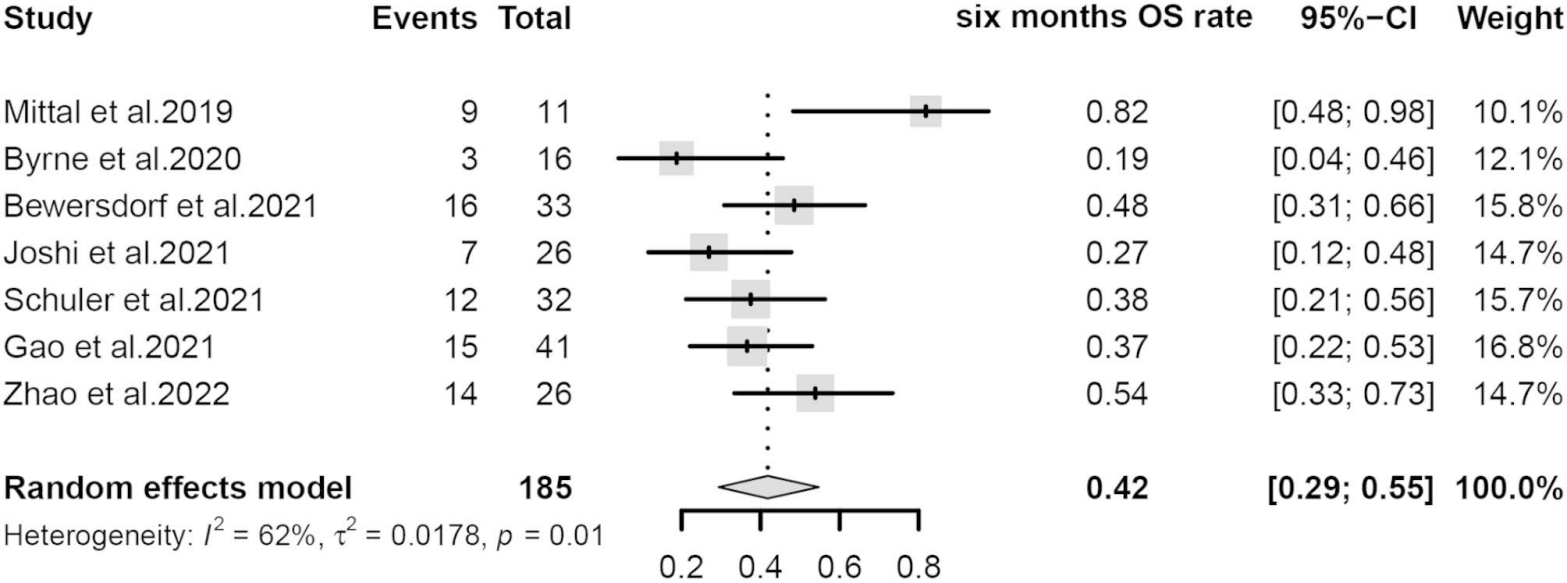
Forest plot of pooled six months OS rates after treatment

**Fig. 5:**
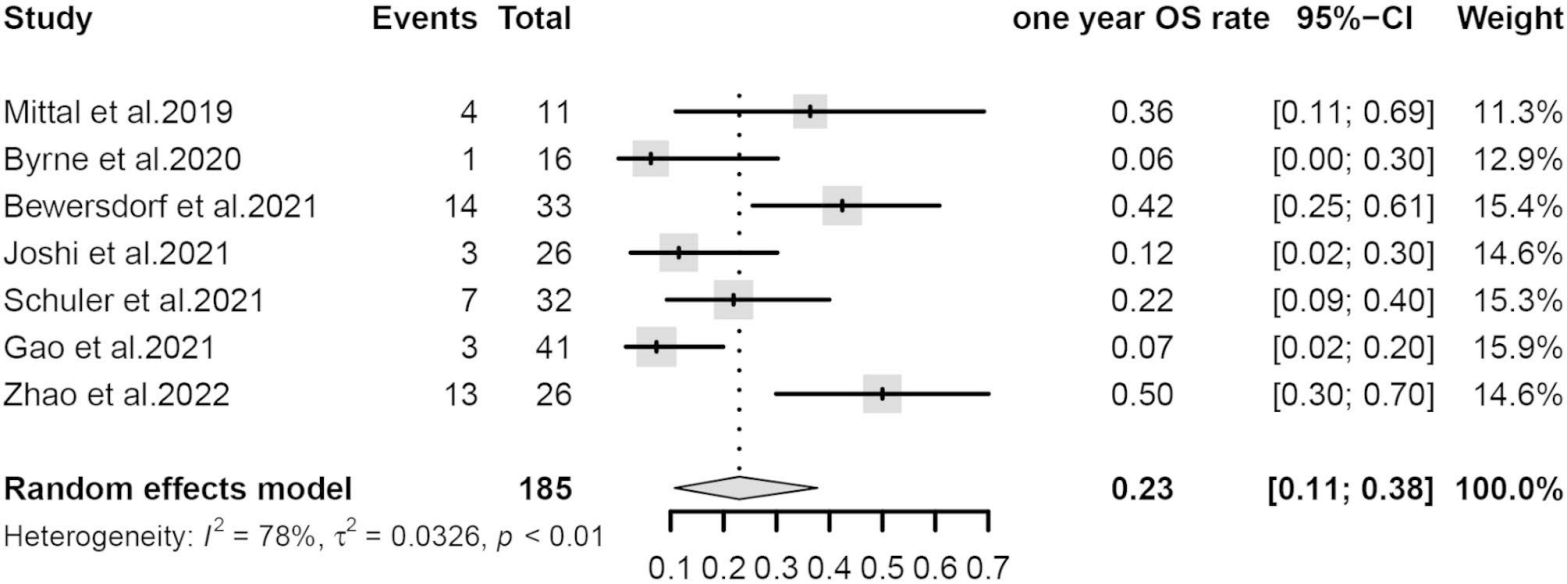
Forest plot of pooled one year OS rates after treatment

The primary outcomes observed in this meta-analysis were CR/CRi, ORR, and Survival rate, which were analyzed using a random-effects model for the included studies. We estimated that the CR/CRi of Ven-HMAs for treating relapse in patients with AML/MDS post-transplantation was 32% (95% CI, 26-39%, I^2^ = 0%; Fig. 2) and the ORR was 48% (95% CI, 39-56%, I^2^ = 37%; Fig. 3), indicating a low heterogeneity. In this study, 6-month survival rate was 42% (95% CI, 29-55%, I^2^ = 62%; Figs. 4) and 1-year survival rate 23% (95% CI, 11-38%, I^2^ = 78%; Fig. 5), respectively. The analysis results in terms of race were as follows: Pooled CR/CRi and ORR in North America were 31% (95%CI, 20-43%, I^2^ = 0%) and 51% (95%CI, 32-70%, I^2^ = 63%), in Europe were 30% (95% CI, 17-45%. I^2^ = 0%) and 42% (95%CI, 27-57%, I^2^ = 0%), and in Asia were 34% (95%CI, 25-45%, I^2^ = 0%) and 51% (95% CI, 37-66%, I^2^ = 45%) (Figure S1-S2).

### AEs of the study

**Fig. 6:**
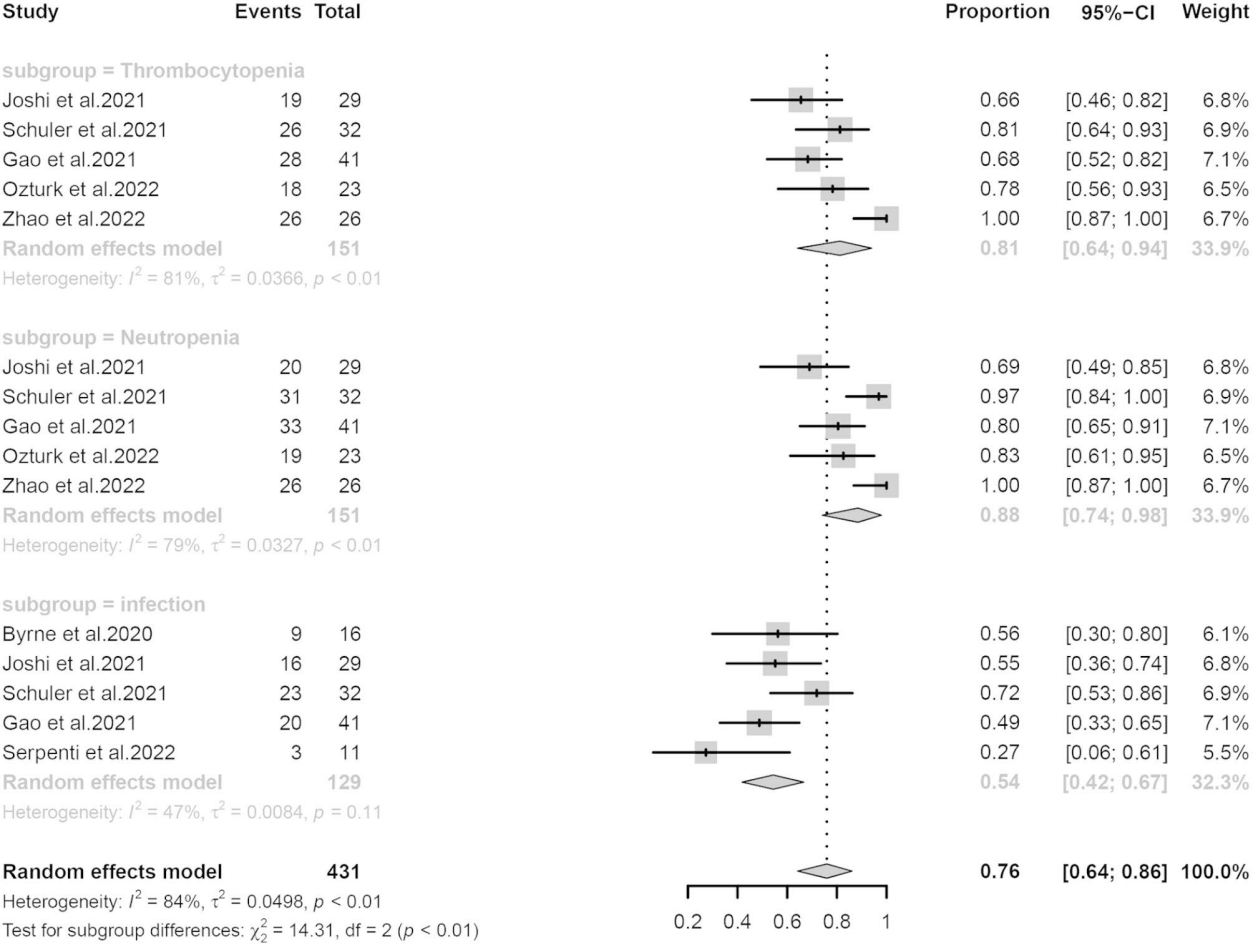
Forest plots of grade 3–4 adverse events: rates of thrombocytopenia, neutropenia, and infection

This study analyzed the incidence of three AEs (grade 3/4) including neutropenia, thrombocytopenia, and infection. Each of these 3 AEs derived from 5 studies was included for further analysis. Specifically, thrombocytopenia was 81% (95% CI, 64-94%, I^2^ = 81%), neutropenia 88% (95% CI, 74-98%, I^2^ = 79%), and infection 54% (95% CI, 42-67%, I^2^ = 47%) (Fig. 6). The three outcomes had moderate to high heterogeneity. Gao et al. reported an an early death in induction related to mortality rates of 11.4% and 22.7% within 30 and 60 days, respectively [30].

### Sensitivity analysis

In this study, sensitivity analyses on the main observed outcomes were performed using the “leave one out” method to further evaluate whether the exclusion of a study would significantly alter the results based on the remaining studies. For a high heterogeneity, potential origins were explored. Sensitivity analysis showed that the results of the meta-analysis of this study were robust (Figure S3-S6).

## Discussion

This meta-analysis included 10 studies of 243 patients who relapsed after receiving AML/MDS transplantation to evaluate the efficacy and safety of the Ven-HMAs. Combined data for the included studies showed that the CR/CRi and ORR of this regimen for managing post-transplantation relapse in AML/MDS were 32% and 48%, respectively, with low heterogeneity. Survival analysis showed a 42% and 23% survival rate at 6 and 12 months, respectively, with a moderate and high heterogeneity. We hypothesized that the heterogeneity might be caused by the heterogeneity of clinical characteristics of patients who relapsed after transplantation (e.g., method of transplantation) on the one hand, on the other hand, the heterogeneity of the number of cycles and duration of treatment after transplantation. For this study, adverse reactions were reported in five articles, and common AEs were thrombocytopenia, neutropenia, and infection. Causes of death during treatment with the Ven-HMAs was not reported in most articles.

B-cell lymphoma-2 (BCL-2) gene was initially regarded as a growth-driven oncogene and was later identified to promote tumor cell survival by inhibiting apoptosis [34, 35]. BCL-2 family proteins are important regulators of the apoptosis signaling pathway. According to protein function, they are divided into anti-apoptotic proteins (BCL-2, BCL-XL, BCL-W, BCL2-A1, MCL-1) and pro-apoptotic proteins (effector proteins: BAX/BAK; BH3-Only proteins: BID, BIK, BIM, BAD, PUMA, NOXA) [36, 37]. The relative levels of anti-apoptotic and pro-apoptotic proteins determine a cell survival or death [38]. High expression of BCL-2 in CD34 + AML cells promotes AML cell survival and is associated with chemotherapy resistance and poor prognosis [39]. Venetoclax has a high affinity for the BH3 binding domain of BCL-2 and promotes apoptosis as well as inhibits cell proliferation by suppressing high BCL-2 expression in AML cells [40, 41]. In 2018, the FDA approved Venetoclax-based therapy for AML, which has witnessed a gradual expansion of its application to the management of post-transplant relapse in AML/MDS in the last three years. This study found that the CR/CRi rate and 1-year survival rate after relapse from AML/MDS transplantation based on Ven-HMAs were 32% and 23%, respectively, which did not seem to show a significant advantage in comparison to the previous HMAs ± DLI regimen [42–46]. The possible reason may be related to different baseline characteristics of the populations included in each study, and the comparative results may be highly inaccurate. On the other hand, the majority (97.5%) of patients included in this study were hematological relapsed and experienced a higher tumor burden than in the previous HMAs ± DLI group, which also influenced the response rate in this study. It should be noticed that more than half of the patients (52.7%) included in this study were previously treated with HMAs and may be insensitive to HMAs alone, however, Ven + HMAs enable some of these patients to achieve CR/CRi again. This also suggested that Ven-HMAs enhanced the anti-leukemic effect, showing a synergistical effect. It is currently believed that the synergistic effect of Venetoclax and HMAs may be through several mechanisms: (1) HMAs induce the expression of the pro-apoptotic protein NOXA through the integrated stress response pathway, which enhances the activation of Venetoclax-induced apoptosis in AML cells [47]; (2) Venetoclax can enhance the sensitivity of AML cells to HMAs by inhibiting the antioxidant response pathway induced by HMAs [48]; (3) Ven-HMAs together impair the tricarboxylic acid cycle of leukemic stem cells (LSCs) and deplete ATP of LSCs leading to AML cell death [49]. Similarly, the CR/CRi rate of relapse after AML/MDS transplantation was low for Ven-HMAs when compared to the previous IC + DLI regimen [11, 50, 51]. In addition to the heterogeneity of the study population, this difference may also be due to inadequate Ven-HMAs combined with cell therapy (only 25% of patients were treated with DLI at the base of Ven-HMAs). Although IC + DLI has a high remission rate, the 2-year survival rate of the patients is not high, which may be associated with a higher rate of treatment-related death (TRM) caused by IC and an inability to maintain a long-term remission [12, 52]. Though TRM was not mentioned explicitly in the studies included in this meta-analysis, previous findings showed that Ven-HMAs did not cause a high rate of TRM in R/R-AML [53, 54]. Therefore, we speculated that Ven-HMAs regimens could be considered as a treatment option for AML/MDS relapsed after transplantation and intolerance to IC (including patients previously treated with HMAs). Relapse < 6 months from transplantation, high tumor burden, poorer karyotype/molecular biology mutations, and aGVHD at the time of relapse have been considered as a poor prognosis of relapse after transplantation in previous studies [8, 45, 55]. However, the study of Bewersdorf et al. suggested that transplantation < 12 months from relapse and *SF3B1* mutation were poor prognostic factors, while mutations in *TP53* were not relevant to response and survival rate [27]. Joshi et al. and Schuler et al. similarly observed that *TP53* mutations were not associated with a poor prognosis in Ven-HMAs treatment [28, 29]. However, the study by Gao et al. found that *TP53* mutations and relapse within 1 year of transplantation were independent risk factors for the outcome, while DNMT3A, NPM1, and IDH1/2 mutations may be associated with favorable CR/CRI rate [30]. It should be noted that the four studies were on a small scale and the prognostic impact of *TP53* mutations still required observational studies based on large samples. A further finding in the study by Schuler et al. showed that only patients with molecular relapse achieved long-term survival from receiving Ven-HMAs therapy. These studies indicated that on one hand, Ven-HMAs regimens may overcome the effects of certain adverse molecular mutations and allow this group of patients to regain remission. On the other hand, it suggested that post-transplantation monitoring was particularly important, especially NGS-based molecular and flow cytometry-based MRD monitoring, and that preemptive treatment at the molecular relapse stage may stimulate a higher response rate to achieve a long-term survival [56–58].

The toxicity of Ven-HMAs regimens is substantial. This study found an incidence of grade 3/4 thrombocytopenia and neutropenia of 81% and 88%, respectively, with a higher incidence than previously reported in untreated AML patients [59, 60]. We hypothesized that the occurrence was related to cytopenia due to disease progression and decreased bone marrow hematopoiesis resulting from the disruption of the hematopoietic microenvironment by previous chemotherapeutic agents [61]. A long-term cytopenia may lead to treatment disruptions and shortened treatment cycles, and whether this would reduce treatment efficacy is unknown. The use of granulocyte-stimulating factors and thrombopoietin to a lower death risk due to cytopenia may also be rational. Granulocyte deficiency could cause a high incidence of infection, and the grade 3/4 infection rate in this study was 54%, which was similar to the results previously reported [53]. However, we speculated that the 54% infection rate was underestimated because some patients used antibiotics (e.g., posaconazole) prophylactically during the treatment period. In this study, one article mentioned that the common cause of death during follow-up was infection and three articles observed that the common cause of death was disease progression [25, 26, 30, 32]. Therefore, monitoring infections during treatment with this regimen was also crucial. Only the study by Gao et al. mentioned early death in induction, with mortality rates of 11.4% and 22.7% within 30 and 60 days, slightly higher than that reported by DiNardo et al. for de novo AML [30, 60]. The reason for this difference may be related to the poor performance status of patients who relapsed after transplantation.

There are several advantages of this meta-analysis. Firstly, a comprehensive search of the available studies revealed that there was no published meta-analysis of Ven-HMAs for AML/MDS relapsed after transplantation. Secondly, we developed a comprehensive search strategy with clear inclusion criteria and strict quality evaluation to ensure the reliability of the current results, and we reported strictly according to PRISMA standards. Finally, the current analysis also evaluated the results of studies from different geographical regions in order to provide a comprehensive reference for future studies.

However, this study inevitably has some limitations. First, the sample size of each study included was small, though we have adequately searched each database. This may be related to the fact that clinical studies of Ven-HMAs in AML/MDS relapse after transplantation were at a preliminary stage. The second, the included clinical studies were retrospective ones and the quality of clinical evidence as prospective studies was not high enough, therefore we hope that prospective studies will emerge to certify the analysis findings in our study. The third, more than half of the studies came from the United States and Asia, and it was unclear whether the current findings can be generalized to other regional populations. The fourthly, a moderate to a high heterogeneity in the data on survival and side effects was present, and we used sensitivity analysis to obtain some factors contributing to the heterogeneity, but a comprehensive analysis on the specific causes to the heterogeneity was difficult. In final, most of the patients included in this study were AML, however, the number of MDS patients included was muchsmall, therefore, more MDS data need to be accumulated to ensure the robustness of the current conclusions.

## Conclusion

In general, this meta-analysis demonstrated a moderate benefit to AML/MDS patients with post-transplant relapses treated with Ven-HMAs regimens, including those who have received prior HMAs therapy. The most common AEs of the regimen were grade 3–4 hemocytopenia and infection. In the future, large prospective clinical studies are needed to validate our research.

### Electronic supplementary material

Below is the link to the electronic supplementary material.

**Supplementary Material 1: Figure S1**. Forest plots of pooled CR/CRi rates with different races.

**Supplementary Material 2: Figure S2**. Forest plots of pooled ORR rates with different races.

**Supplementary Material 3: Figure S3**. Sensitivity analysis of pooled CR/CRi rates after treatment.

**Supplementary Material 4: Figure S4**. Sensitivity analysis of pooled ORR rates after treatment.

**Supplementary Material 5: Figure S5**. Sensitivity analysis of pooled six months OS rates after treatment.

**Supplementary Material 6: Figure S6**. Sensitivity analysis of pooled one year OS rates after treatment.

**Supplementary Material 7: Table S1**. The strategy of literature search (from inception to 23 February 2023)

**Supplementary Material 8: Table S2**. The quality of the 10 included studies was assessed by MINORS.

## Data Availability

This article and supplemental material included all the data generated during this study. For further inquiries, please contact the corresponding author.
