# Supplementary figures and images for: Efficacy and safety of venetoclax combined with hypomethylating agents for relapse of acute myeloid leukemia and myelodysplastic syndrome post allogeneic hematopoietic stem cell transplantation: a systematic review and meta-analysis
Source: BMC Cancer. 2023 Aug 17;23:764. doi: 10.1186/s12885-023-11259-6 (PMC10433628; doi:10.1186/s12885-023-11259-6)

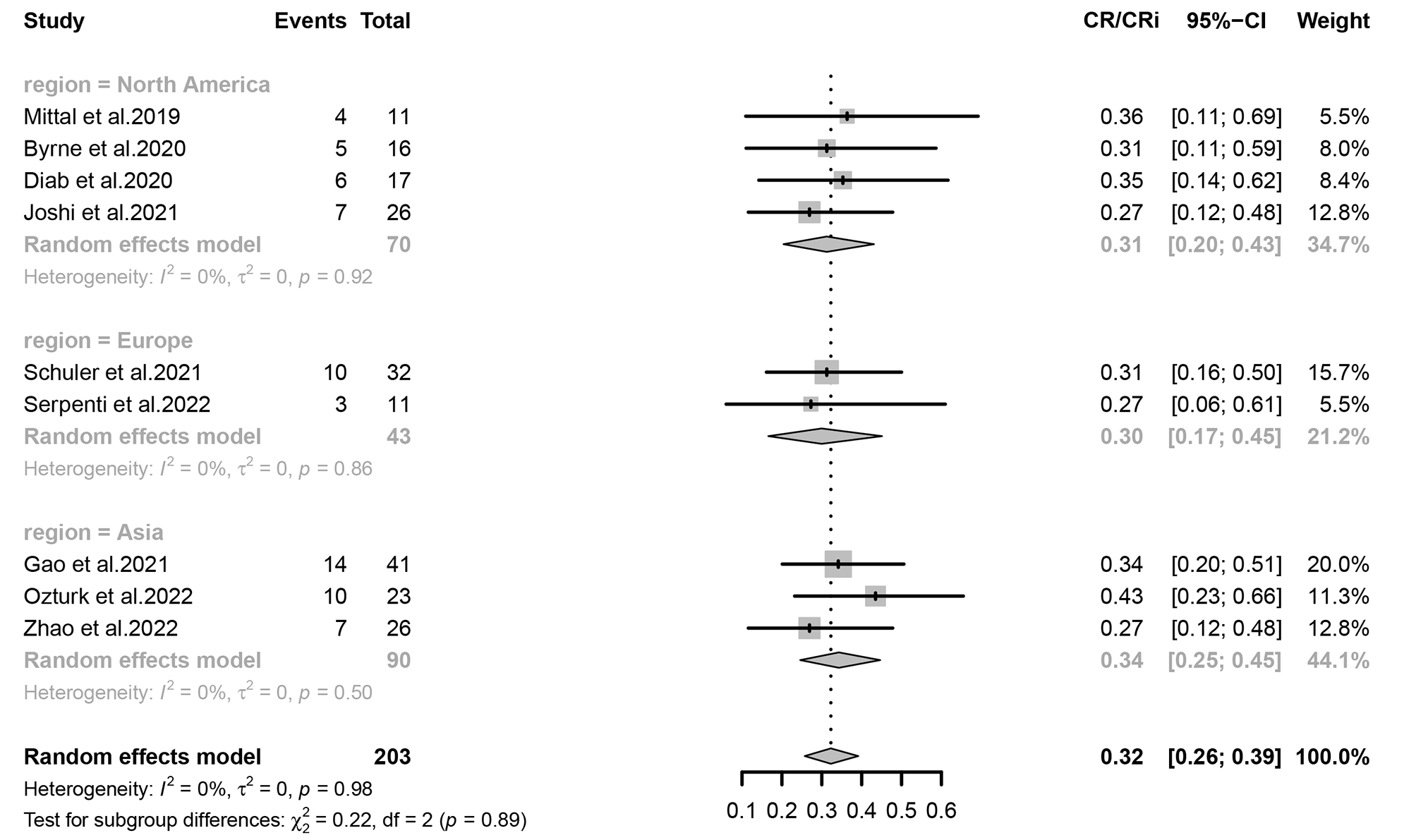

Supplement: Supplementary file 1 — Supplementary Material 1: Figure S1. Forest plots of pooled CR/CRi rates with different races. [file 12885_2023_11259_MOESM1_ESM.png]

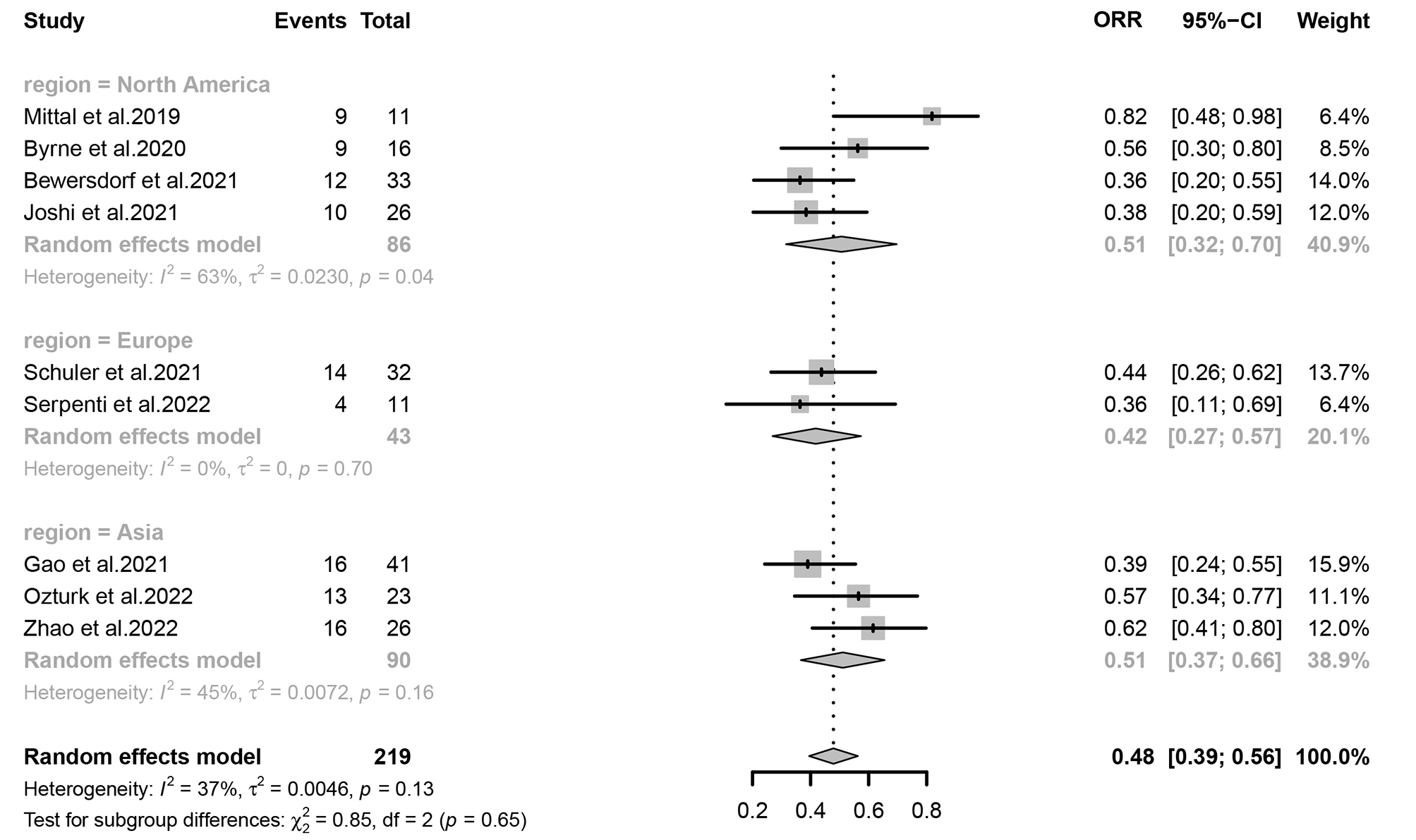

Supplement: Supplementary file 2 — Supplementary Material 2: Figure S2. Forest plots of pooled ORR rates with different races. [file 12885_2023_11259_MOESM2_ESM.png]

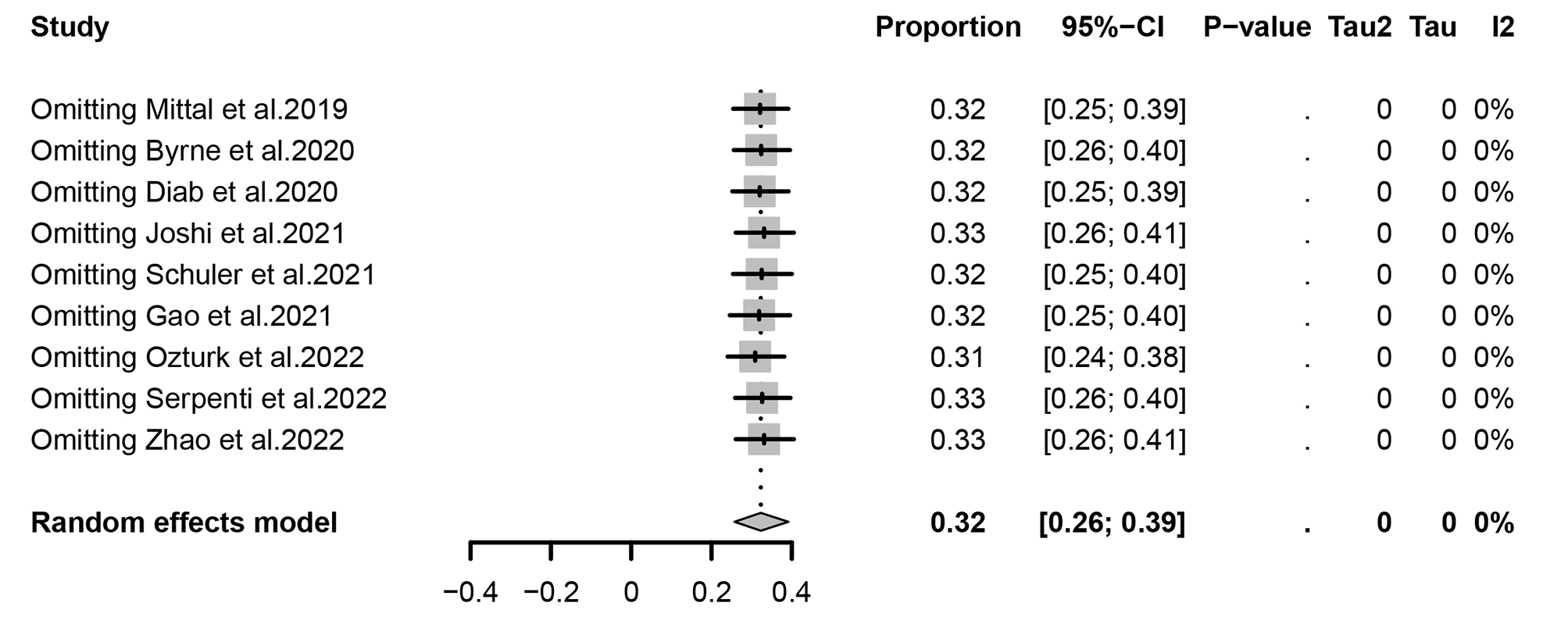

Supplement: Supplementary file 3 — Supplementary Material 3: Figure S3. Sensitivity analysis of pooled CR/CRi rates after treatment. [file 12885_2023_11259_MOESM3_ESM.png]

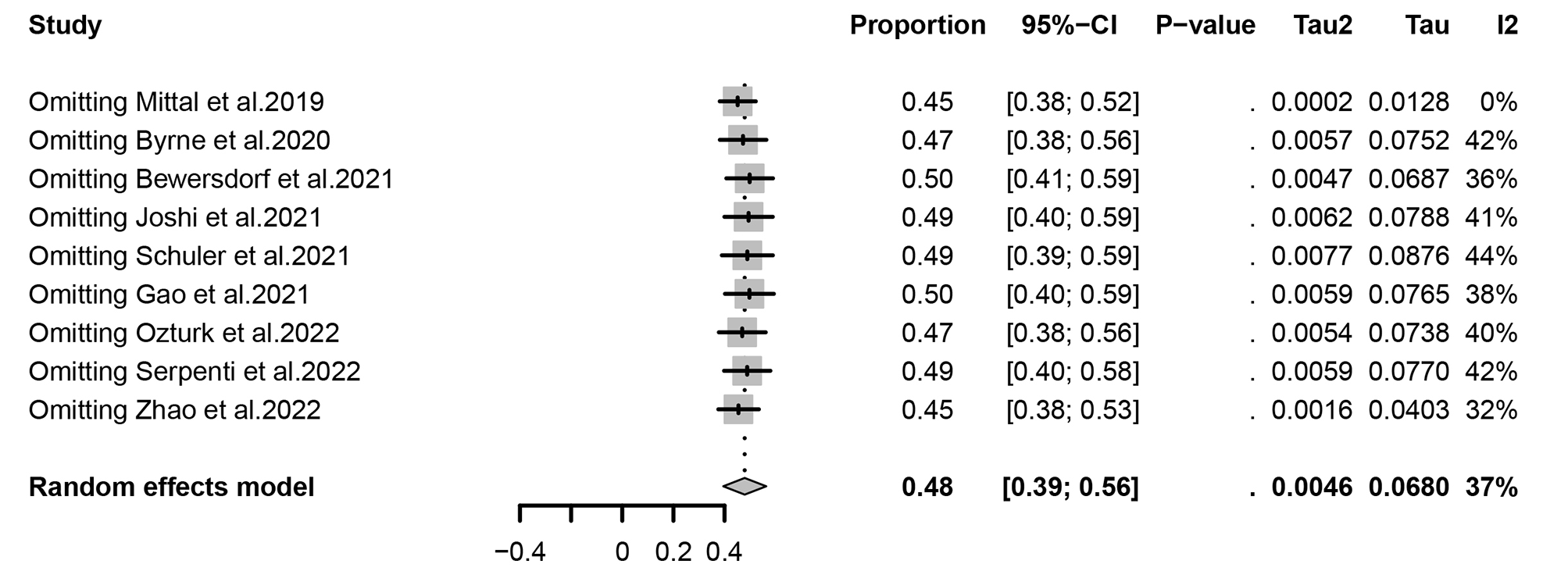

Supplement: Supplementary file 4 — Supplementary Material 4: Figure S4. Sensitivity analysis of pooled ORR rates after treatment. [file 12885_2023_11259_MOESM4_ESM.png]

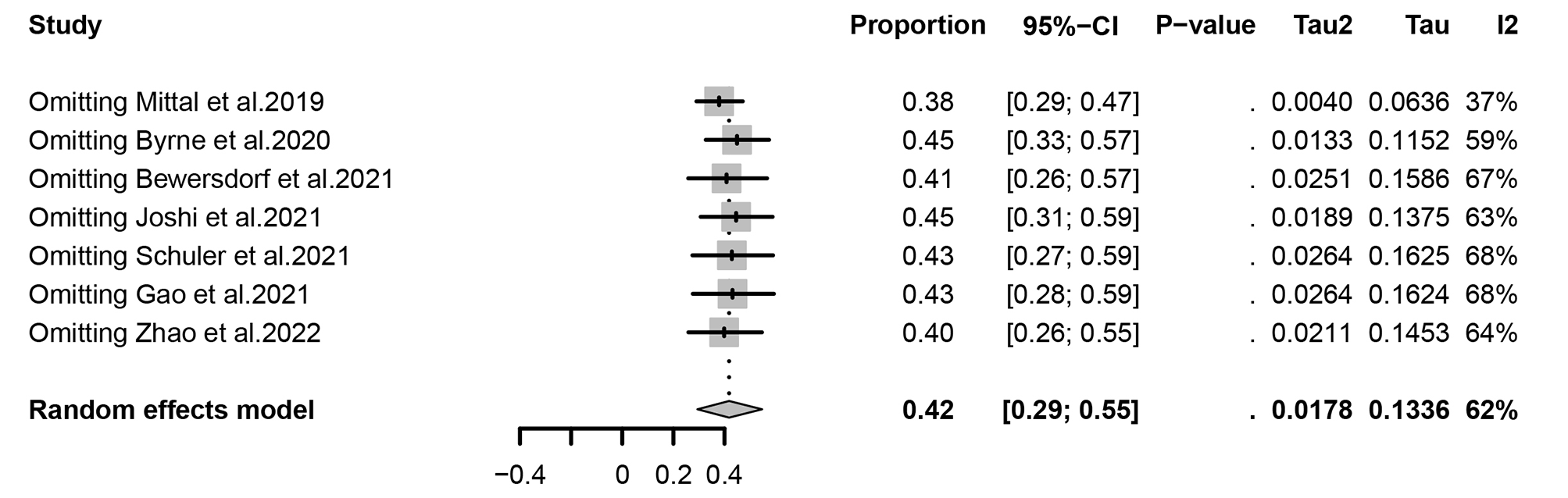

Supplement: Supplementary file 5 — Supplementary Material 5: Figure S5. Sensitivity analysis of pooled six months OS rates after treatment. [file 12885_2023_11259_MOESM5_ESM.png]

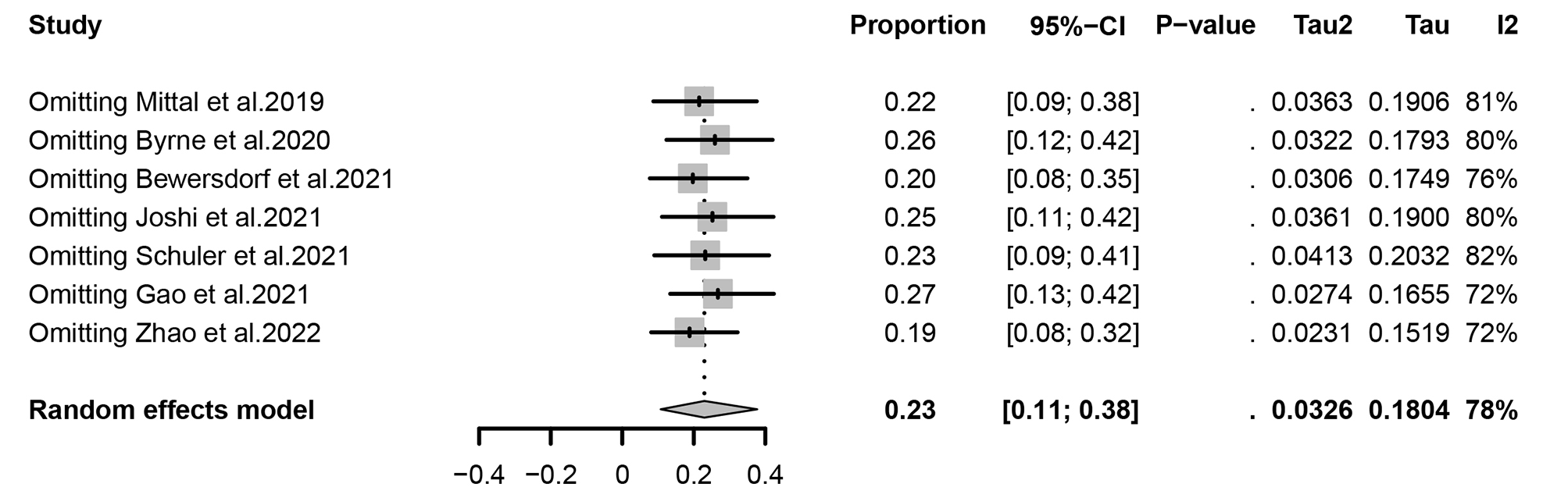

Supplement: Supplementary file 6 — Supplementary Material 6: Figure S6. Sensitivity analysis of pooled one year OS rates after treatment. [file 12885_2023_11259_MOESM6_ESM.png]
