# Supplementary material for: Efficacy and safety of venetoclax combined with hypomethylating agents for relapse of acute myeloid leukemia and myelodysplastic syndrome post allogeneic hematopoietic stem cell transplantation: a systematic review and meta-analysis
Source: BMC Cancer. 2023 Aug 17;23:764. doi: 10.1186/s12885-023-11259-6 (PMC10433628; doi:10.1186/s12885-023-11259-6)
Supplement: Supplementary file 7 — Supplementary Material 7: Table S1. The strategy of literature search (from inception to 23 February 2023) [file 12885_2023_11259_MOESM7_ESM.docx]

Table S1. The strategy of literature search (from inception to 23 February 2023).

| Databases | Search string | Results |
| --- | --- | --- |
| PubMed | #1 "venetoclax"[Supplementary Concept] | 1284 |
|  | #2 "venetoclax"[Title/Abstract] OR "GDC-0199"[Title/Abstract] OR "RG7601"[Title/Abstract] OR "Venclexta"[Title/Abstract] OR "ABT-199"[Title/Abstract] | 2424 |
|  | #3 (#1) OR (#2) | 2357 |
|  | #4 "azacitidine"[MeSH Terms] | 7879 |
|  | #5 "azacitidine"[Title/Abstract] OR "Azacytidine"[Title/Abstract] OR "Vidaza"[Title/Abstract] OR "nsc 102816"[Title/Abstract] | 5651 |
|  | #6(#4) OR (#5) | 10111 |
|  | #7 "decitabine"[MeSH Terms] | 3776 |
|  | #8 "decitabine*"[Title/Abstract] OR "2 deoxy 5 azacytidine"[Title/Abstract] OR "5-Deoxyazacytidine"[Title/Abstract] OR "Dacogen"[Title/Abstract] OR "azadc compound"[Title/Abstract] OR "5 aza*"[Title/Abstract] OR "NSC-127716"[Title/Abstract] | 10931 |
|  | #9(#7) OR (#8) | 11476 |
|  | #10 "hypomethylating agent*"[Title/Abstract] OR "HMA"[Title/Abstract] | 3166 |
|  | #11 (#6) OR (#9) OR (#10) | 15764 |
|  | #12 "leukemia, myeloid, acute"[MeSH Terms] | 63198 |
|  | #13 "acut*"[Title/Abstract] OR "akut*"[Title/Abstract] OR "agud*"[Title/Abstract] OR "aigu*"[Title/Abstract] | 1444148 |
|  | #14 ("myelo*"[Title/Abstract] OR "nonlympho*"[Title/Abstract] OR "mielo*"[Title/Abstract]) AND ("leukaem*"[Title/Abstract] OR "leuc*"[Title/Abstract]) | 18697 |
|  | #15 (#13) and (#14) | 9999 |
|  | #16 "acute myeloid leukemia"[Title/Abstract] OR "Aml"[Title/Abstract] OR "anll"[Title/Abstract] | 53993 |
|  | #17 (#12) OR (#15) OR (#16) | 90588 |
|  | #18 "myelodysplastic syndromes"[MeSH Terms] | 23369 |
|  | #19 "myeloplasti*"[Title/Abstract] OR "dysmyelopoietic*"[Title/Abstract] OR "myelodysplas*"[Title/Abstract] | 23606 |
|  | #20 "Mds"[Title/Abstract] | 21323 |
|  | #21 "anemia, refractory"[MeSH Terms] | 1753 |
|  | #22 ("refrakta*"[Title/Abstract] OR "refract*"[Title/Abstract]) AND ("anem*"[Title/Abstract] OR "anaem*"[Title/Abstract]) | 6579 |
|  | #23 (#18) OR (#19) OR (#20) OR (#21) OR (#22) | **45395** |
|  | #24 "stem cell transplantation"[MeSH Terms] | 97393 |
|  | #25 "SCT"[Title/Abstract] OR "BMT"[Title/Abstract] OR "PBSC"[Title/Abstract] OR "PSCT"[Title/Abstract] OR "BMCT"[Title/Abstract] OR "BM"[Title/Abstract] | 54976 |
|  | #26 "stem cell transplantation"[Title/Abstract] OR "bone marrow transplantation"[Title/Abstract] OR "peripheral blood stem cells transplantation"[Title/Abstract] | 76677 |
|  | #27(#24) OR (#25) OR (#26) | 173985 |
|  | #28 "recurrence"[MeSH Terms] | 199331 |
|  | #29 "recurrence*"[Title/Abstract] OR "recrudescence*"[Title/Abstract] OR "relapse*"[Title/Abstract] | 553760 |
|  | #30 (#28) OR (#29) | 668563 |
|  | #31 (#3) and (#11) | 507 |
|  | #32 (#17) OR (#23) | 125480 |
|  | #33 (#27) and (#32) | 16390 |
|  | #34 (#30) and (#33) | 6617 |
|  | #35 (#11) OR (#34) | 44 |
| EMBASE | #1 venetoclax'/exp | 8789 |
|  | #2 venetoclax:ab,ti,kw OR 'gdc-0199':ab,ti,kw OR 'rg7601':ab,ti,kw OR 'venclexta':ab,ti,kw OR 'abt-199':ab,ti,kw | 6434 |
|  | #3 #1 OR #2 | 9250 |
|  | #4 ‘azacitidine'/exp | 17975 |
|  | #5 azacitidine':ab,ti,kw OR 'azacytidine':ab,ti,kw OR 'vidaza':ab,ti OR 'nsc 102816':ab,ti,kw | 11366 |
|  | #6 #4 OR #5 | 19248 |
|  | #7 'decitabine'/exp | 15125 |
|  | #8 decitabine*:ti,ab,kw OR '5 aza*':ti,ab,kw OR 'azadc compound':ti,ab,kw OR '2 deoxy 5 azacytidine':ti,ab,kw OR '5 deoxyazacytidine':ti,ab,kw OR dacogen:ti,ab,kw OR 'nsc 127716':ti,ab,kw | 17,528 |
|  | #9 (#7) OR (#8) | 23159 |
|  | #10 'hypomethylating agent'/exp | 200 |
|  | #11 'hypomethylating agent*' ti,ab,kw | 5438 |
|  | #12 #10 OR #11 | 5496 |
|  | #13 #6 OR #9 OR #12 | 33672 |
|  | #14 #3 AND #13 | 2452 |
|  | #15 'acute myeloid leukemia'/exp | 130862 |
|  | #16 acut*:ti,ab,kw OR akut*:ti,ab,kw OR agud*:ti,ab,kw OR aigu*:ti,ab,kw | 2,061,660 |
|  | #17 myelo*:ti,ab,kw OR nonlympho*:ti,ab,kw OR mielo*:ti,ab,kw | 470,045 |
|  | #18 leukaem*:ti,ab,kw OR leuc*:ti,ab,kw | 219979 |
|  | #19 #16 AND #17 AND #18 | 15653 |
|  | #20 'acute myeloid leukemia':ti,ab,kw OR aml:ti,ab,kw OR anll:ti,ab,kw | 101,089 |
|  | #21 #15 OR #19 OR #20 | 163333 |
|  | #22 'myelodysplastic syndrome'/exp | 52,436 |
|  | #23 'myelodysplastic syndrome':ti,ab,kw OR myeloplasti*:ti,ab,kw OR dysmyelopoietic*:ti,ab,kw OR myelodysplas*:ti,ab,kw | 41,482 |
|  | #24 mds:ti,ab,kw | 45648 |
|  | #25 'refractory anemia'/exp | 2788 |
|  | #26 refrakta*:ti,ab,kw OR refract*:ti,ab,kw | 327734 |
|  | #27 anem*:ti,ab,kw OR anaem*:ti,ab,kw | 272226 |
|  | #28 #26 AND #27 | 14776 |
|  | #29 'refractory anemia':ti,ab,kw | 3189 |
|  | #30 #22 OR #23 OR #24 OR #25 OR #28 OR #29 | 89806 |
|  | #31 #21 OR #30 | 219516 |
|  | #32 'stem cell transplantation'/exp | 185107 |
|  | #33 'stem cell transplantation':ti,ab,kw OR 'bone marrow transplantation':ti,ab,kw OR 'eripheral blood stem cell transplantation':ti,ab,kw | 127,638 |
|  | #34 sct:ti,ab,kw OR bmt:ti,ab,kw OR pbsc:ti,ab,kw OR psct:ti,ab,kw OR bmct:ti,ab,kw | 44,937 |
|  | #35 #32 OR #33 OR #34 | 243,950 |
|  | #36 'recurrent disease'/exp | 210700 |
|  | #37 'recurrent disease':ti,ab,kw OR recurrence*:ti,ab,kw OR recrudescence*:ti,ab,kw OR relapse*:ti,ab,kw | 895,586 |
|  | #38 #36 OR #37 | 1009841 |
|  | #39 #14 AND #31 AND #35 AND #38 | 435 |
| CENTRAL | #1 (GDC 0199):ti,ab,kw OR (RG 7601):ti,ab,kw OR (ABT 199):ti,ab,kw OR (Venclexta):ti,ab,kw OR (venetoclax):ti,ab,kw (Word variations have been searched) | 551 |
|  | #2 MeSH descriptor: [Azacitidine] explode all trees | 453 |
|  | #3 (Azac?tidine):ti,ab,kw OR (Vidaza):ti,ab,kw OR (NSC 102816):ti,ab,kw | 1096 |
|  | #4 #2 OR #3 | 1148 |
|  | #6 (Decitabine):ti,ab,kw OR (5 Aza):ti,ab,kw OR (Azadc compound):ti,ab,kw OR (5 Deoxyazacytidine):ti,ab,kw OR (NSC 127716):ti,ab,kw | 1530 |
|  | #7 #5 OR #6 | 1530 |
|  | #8 (Hypomethylating agent):ti,ab,kw | 259 |
|  | #9 #4 OR #7 OR #8 | 2191 |
|  | #10 #1 AND #9 | 141 |
|  | #11 MeSH descriptor: [Leukemia, Myeloid, Acute] explode all trees | 1952 |
|  | #12 (acut*):ti,ab,kw OR (akut*):ti,ab,kw OR (agud*):ti,ab,kw OR (aigu*):ti,ab,kw | 163365 |
|  | #13 (myelo*):ti,ab,kw OR (nonlympho*):ti,ab,kw OR (mielo*):ti,ab,kw | 21337 |
|  | #14 (leukaem*):ti,ab,kw OR (leuc*):ti,ab,kw | 11415 |
|  | #15 #12 AND #13 AND #14 | 983 |
|  | #16 (acute myeloid leukemia):ti,ab,kw OR (AML):ti,ab,kw OR (ANLL):ti,ab,kw | 6694 |
|  | #17 #11 OR #15 OR #16 | 6976 |
|  | #18 MeSH descriptor: [Myelodysplastic Syndromes] explode all trees | 851 |
|  | #19 (myeloplasti*):ti,ab,kw OR (dysmyelopoietic*):ti,ab,kw OR (myelodysplas*):ti,ab,kw | 2718 |
|  | #20 (MDS):ti,ab,kw | 3310 |
|  | #21 #18 OR #19 OR #20 | 4602 |
|  | #22 MeSH descriptor: [Anemia, Refractory] explode all trees | 50 |
|  | #23 (refrakta*):ti,ab,kw OR (refract*):ti,ab,kw | 26783 |
|  | #24 (anem*):ti,ab,kw OR (anaem*):ti,ab,kw | 23854 |
|  | #25 #23 AND #24 | 1513 |
|  | #26 #22 OR #25 | 1513 |
|  | #27 #21 OR #26 | 5755 |
|  | #28 #17 OR #27 | 10794 |
|  | #29 MeSH descriptor: [Stem Cell Transplantation] explode all trees | 2855 |
|  | #30 (stem cell transplantation):ti,ab,kw OR (bone marrow transplantation):ti,ab,kw OR (Peripheral blood stem cell transplantation):ti,ab,kw | 12275 |
|  | #31 (SCT):ti,ab,kw OR (BMT):ti,ab,kw OR (BMCT):ti,ab,kw OR (PBSC):ti,ab,kw OR (PSCT):ti,ab,kw | 3719 |
|  | #32 #29 OR #30 OR #31 | 14032 |
|  | #33 MeSH descriptor: [Recurrence] explode all trees | 14363 |
|  | #34 (Recurrence):ti,ab,kw OR (Recrudescence):ti,ab,kw OR (relapse):ti,ab,kw | 79672 |
|  | #35 #33 OR #34 | 79795 |
|  | #36 #10 AND #28 AND #32 AND #35 | 21 |
| Web of science | #1 venetoclax (Topic) OR GDC-0199 (Topic) OR RG7601 (Topic) OR ABT-199 (Topic) OR Venclexta (Topic) | 4,051 |
|  | #2 Azacitidine (Topic) or Vidaza (Topic) or NSC 102816 (Topic) or Azacytidine (Topic) | 8638 |
|  | #3 Decitabine* (Topic) or 5-Aza* (Topic) or AzadC compound (Topic) or 2'-Deoxy-5-azacytidine (Topic) or 5-Deoxyazacytidine (Topic) or Dacogen (Topic) or NSC-127716 (Topic) | 14199 |
|  | #4 Hypomethylating agent* (Topic) or HMA (Topic) | 7229 |
|  | #5 #2 OR #3 OR #4 | 23059 |
|  | #6 #1 OR #5 | 784 |
|  | #7 acut* (Topic) or akut* (Topic) or agud* (Topic) or aigu* (Topic) | 1608787 |
|  | #8 myelo* (Topic) or nonlympho* (Topic) or mielo* (Topic) | 401416 |
|  | #9 leukaem* (Topic) or leuc* (Topic) | 206801 |
|  | #10 #7 and #8 and #9 | 10538 |
|  | #11 leukemia, myeloid, acute (Topic) or Aml (Topic) or anll (Topic) | 102586 |
|  | #12 #10 OR #11 | 104136 |
|  | #13 myelodysplastic syndromes (Topic) or myeloplasti* (Topic) or dysmyelopoietic* (Topic) or myelodysplas* (Topic) | 35665 |
|  | #14 Mds (Topic) | 33307 |
|  | #15 anemia, refractory (Topic) | 6471 |
|  | #16 refrakta* (Topic) or refract* (Topic) | 431516 |
|  | #17 anem* (Topic) or anaem* (Topic) | 208632 |
|  | #18 #16 and #17 | 6881 |
|  | #19 #13 OR #14 OR #15 OR #18 | 60840 |
|  | #20 #12 OR #19 | 148653 |
|  | #21 Stem cell transplantation (Topic) or bone marrow transplantation (Topic) or Peripheral blood stem cell transplantation (Topic) | 193560 |
|  | #22 SCT (Topic) or BMT (Topic) or PBSC (Topic) or PSCT (Topic) or BMCT (Topic) | 32166 |
|  | #23 #21 OR #22 | 208563 |
|  | #24 Recurrence* (Topic) or Recrudescence* (Topic) or Relapse* (Topic) | 601251 |
|  | #25 #6 and #20 and #23 and #24 | 49 |
