# Supplementary material for: Efficacy and safety of venetoclax combined with hypomethylating agents for relapse of acute myeloid leukemia and myelodysplastic syndrome post allogeneic hematopoietic stem cell transplantation: a systematic review and meta-analysis
Source: BMC Cancer. 2023 Aug 17;23:764. doi: 10.1186/s12885-023-11259-6 (PMC10433628; doi:10.1186/s12885-023-11259-6)
Supplement: Supplementary file 8 — Supplementary Material 8: Table S2. The quality of the 10 included studies was assessed by MINORS. [file 12885_2023_11259_MOESM8_ESM.docx]

TABLE S2. The quality of the 10 included studies was assessed by MINORS.

| Study | A clearly stated aim | Inclusion of consecutive patients | Prospective collection of data | Endpoints appropriate to the aim of the study | Unbiased assessment of the study endpoint | Follow-up period appropriate to the aim of the study | Loss to follow up less than 5% | Prospective calculation of the study size | An adequate control group | Contemporary groups | Baseline equivalence of groups | Adequate statistical analyses | Scores |
| --- | --- | --- | --- | --- | --- | --- | --- | --- | --- | --- | --- | --- | --- |
| Mittal. et al | 2 | 2 | 2 | 2 | 0 | 2 | 2 | 0 | - | - | - | - | 12 |
| Byrne. et al | 2 | 2 | 2 | 2 | 0 | 2 | 2 | 0 | - | - | - | - | 12 |
| Diab.et al | 2 | 2 | 2 | 2 | 0 | 2 | 2 | 0 | - | - | - | - | 12 |
| Bewersdorf. et al | 2 | 2 | 2 | 2 | 0 | 2 | 2 | 0 | - | - | - | - | 12 |
| Joshi.et al | 2 | 2 | 2 | 2 | 0 | 2 | 2 | 0 | - | - | - | - | 12 |
| Schuler. et al | 2 | 2 | 2 | 2 | 0 | 2 | 2 | 0 | - | - | - | - | 12 |
| Gao.et al | 2 | 2 | 2 | 2 | 0 | 2 | 2 | 0 | - | - | - | - | 12 |
| Ozturk. et al | 2 | 2 | 2 | 2 | 0 | 2 | 2 | 0 | - | - | - | - | 12 |
| Serpenti. et al | 2 | 2 | 2 | 2 | 0 | 2 | 2 | 0 | - | - | - | - | 12 |
| Zhao. et al | 2 | 2 | 2 | 2 | 0 | 2 | 2 | 0 | - | - | - | - | 12 |

Note: The items are scored as 0 (not reported), 1 (reported but inadequate), or 2 (reported and adequate). -, No control group.
